# The Association between the PR Interval and Left Ventricular Measurements in the Multiethnic Study of Atherosclerosis

**DOI:** 10.1155/2015/193698

**Published:** 2015-10-19

**Authors:** Michael P. Husby, Elsayed Z. Soliman, Jeffrey J. Goldberger, Kiang Liu, Don Lloyd-Jones, Ramon Durazo-Arvizu, Holly Kramer

**Affiliations:** ^1^Department of Public Health Sciences, Loyola University Chicago, Maywood, IL 60153, USA; ^2^Department of Medicine, Wake Forest University School of Medicine, Winston Salem, NC 27157, USA; ^3^Department of Preventive Medicine, Northwestern University Feinberg School of Medicine, Chicago, IL 60611, USA

## Abstract

*Introduction.* Few studies have examined the association between the PR interval (PRi) and subclinical cardiovascular disease measures. *Methods and Results*. The Multiethnic Study of Atherosclerosis (MESA) is a population-based study of 6814 men and women aged 45–84 years without clinical cardiovascular disease and 4962 had complete baseline data on cardiac magnetic resonance imaging measures of LV dimension and ejection fraction and surface electrocardiogram. Linear regression models were constructed to determine the adjusted association between the PRi and measures of LV stroke volume, LV mass, LV end-systolic and end-diastolic volumes, and ejection fraction. Overall, mean age was 61.5 years, and 47.6% were male and race/ethnicity was white in 39.1%, Chinese in 13.1%, African-American in 25.7%, and Hispanic in 22.2%. The PRi ranged from 88 to 308 ms with a median value of 162 ms. As a continuous variable, every standard deviation unit (25 ms) increment in PRi was associated with a 2.00 mL (95% CI 1.52, 2.48) higher stroke volume, a 3.08 g (95% CI 2.30, 3.86) higher LV mass, a 1.36 g/m^2^ (95% CI 0.96, 1.76) higher LV mass index, and 1.31 mL (95% CI 0.88, 1.73) higher end-systolic and 3.31 mL (95% CI 2.58, 4.03) higher end-diastolic volumes after adjustment for all covariates. No significant association was noted between the PRi and LV ejection fraction. *Conclusions.* A prolonged PRi is associated with LV measures and may in part explain the link between a prolonged PRi and cardiovascular outcomes.

## 1. Introduction

The slowing of conduction through the AV node may be assessed by the PR interval (PRi) on a surface electrocardiogram. The PRi reflects the time, measured in milliseconds (ms), for the electrical impulse to travel from the sinoatrial node to the atrioventricular node and to the Purkinje fibers [1] or time from onset of atrial depolarization to beginning of ventricular depolarization. Normally, the PRi ranges from 120 to 200 ms and intervals > 200 ms define a prolonged Pri [2]. Historically, a prolonged PRi by itself, in the absence of other conduction abnormalities, was believed to not progress to other forms of heart block [3]. Thus, presence of a prolonged PRi did not indicate a need for treatment other than correcting any electrolyte abnormalities or removing offending drugs [4, 5]. However, several recent studies have suggested that a shortened or prolonged PRi may indicate heightened risk for cardiovascular outcomes including congestive heart failure, atrial fibrillation, and mortality but most of these studies focused on adults with established cardiovascular disease [1, 6–11].

The PRi reflects the timing between atrial and ventricular systole and a longer period of ventricular filling will lead to higher stroke volumes and ventricular wall stress [11], heightening risk for future cardiovascular disease. The importance of the PRi is illustrated by right ventricular (RV) pacing, which increases risk of worsening LV function over time [12–14]. The objective of this study is to utilize data from the Multiethnic Study of Atherosclerosis, a well characterized cohort of adults without clinical cardiovascular disease or active implantable cardiac device at baseline, to examine the association between the PRi and LV dimensions and ejection fraction. We hypothesize that a prolonged PRi is associated with higher LV stroke volume and a lower ejection fraction among adults without established cardiovascular disease.

## 2. Methods

### 2.1. Study Population

The Multiethnic Study of Atherosclerosis (MESA) is a population-based study of 6814 men and women aged 45–84 years, without clinical cardiovascular disease, recruited from six US communities (Baltimore, MD; Chicago, IL; Forsyth County, NC; Los Angeles County, CA; Northern Manhattan, NY; and St. Paul, MN). The main objective of the MESA Study is to determine the characteristics of subclinical cardiovascular disease and its progression. Sampling and recruitment procedures have been previously described in detail [12]. Subjects with symptoms or history of medical or surgical treatment for cardiovascular disease were excluded. During the recruitment process, potential participants were asked about their race/ethnicity. Questions on race/ethnicity were based on the US 2000 census questionnaire. Subjects who self-reported their race/ethnicity group as white or Caucasian, black or African-American, Chinese, or Spanish/Hispanic/Latino were asked to participate. Race/ethnicity was then categorized as white (non-Hispanic), black (non-Hispanic), Chinese, and Hispanic. Subjects were enrolled between 12/1/00 and 7/30/02. Adults weighing >300 pounds and participants with pacemakers and ECG-diagnosed atrial fibrillation/flutter were not eligible for participation. The institutional review boards at all participating centers approved the study, and all participants gave informed consent. A total of 57 participants with missing surface electrocardiogram were excluded along with 2 individuals with a PRi > 320 ms. An additional 1793 participants who did not undergo an MRI were excluded leaving a total of 4962 included in the analysis. Sensitivity analyses were completed after excluding MESA participants (*n* = 967) using medications that may impact the PRi (calcium channel blockers, beta blockers, digoxin, and any antiarrhythmic medications).

### 2.2. PR Interval

Three sequential 10-second resting 12-lead ECGs were digitally acquired using a GE/Marquette MAC-PC electrocardiograph (Marquette Electronics, Milwaukee, Wisconsin) at 10 mm/mV calibration and speed of 25 mm/sec. All ECGs were centrally read and visually inspected for technical errors and inadequate quality at the Epidemiological Cardiology Research Center (EPICARE), Wake Forest School of Medicine (Winston-Salem, NC). A prolonged PRi was defined as a PRi > 200 ms. A shortened PRi was defined as a PRi < 120 ms.

### 2.3. Left Ventricular Mass Index, Dimensions, and Ejection Fraction

Participants underwent a cardiac MRI scan within a median of 16 days after the baseline evaluation and 95% were completed by 11 weeks after the baseline examination. The MESA cardiac MRI protocol, image analysis, and inter- and intrareader reproducibility have been previously reported [15]. Briefly, LV mass, volumes, and functional parameters were determined from short-axis fast gradient echo cine images covering the heart from base to apex throughout the cardiac cycle with temporal resolution ≤50 ms. LV mass was determined by the sum of the myocardial area (the difference between endocardial and epicardial contour) multiplied by the slice thickness plus image gap in the end-diastolic phase multiplied by the specific gravity of myocardium (1.05 g/mL). LV mass was examined with and without indexing for body surface area [16]. LV end-diastolic volume and LV end-systolic volume were calculated using Simpson's rule (the summation of areas on each separate slice multiplied by the sum of slice thickness and image gap). LV stroke volume was calculated as the difference between LV end-diastolic volume and LV end-systolic volume. LV ejection fraction was calculated as LV stroke volume divided by LV end-diastolic volume multiplied by 100 [15].

### 2.4. Covariates

All MESA participants completed self-administered questionnaires, provided fasting blood samples, and were interviewed and examined by trained research staff. Self-administered questionnaires were available in English, Spanish, and Chinese. Resting blood pressure and heart rate were measured 3 times with participants in the seated position with a Dinamap model Pro 100 automated oscillometric sphygmomanometer (Critikon, GE Healthcare, Waukesha, Wisconsin). The average of the last 2 measurements was used for the analysis. Presence of diabetes was defined as self-reported physician diagnosis, use of insulin or oral hypoglycemic agents, or fasting glucose ≥ 126 mg/dL. Current smoking status was based on self-report. Participants were instructed to bring in all existing medications, which were then recorded by research staff. Use of antihypertensive medication was defined as self-reported treatment for hypertension with one of six common classes of antihypertensive medications (thiazide diuretics, beta blockers, calcium channel blockers, angiotensin converting enzyme inhibitors (ACEi), angiotensin-2 receptor blockers (ARB), and other (alpha blockers or peripheral vasodilators)).

### 2.5. Statistical Analysis

Histograms were created to assess the shape of the distribution of the PRi and LV measures among the MESA participants included in the analyses. Scatterplots of PRi by LV end-diastolic volume, LV end-systolic volume, LV stroke volume, LV mass and LV mass index, and ejection fraction were examined. Spearman rank correlation coefficients between PRi and the LV measures and ejection fraction were calculated. Summary statistics for key baseline characteristics were compared by PRi categories. Continuous variables were compared using ANOVA and categorical variables were compared using the Fishers exact test. If these tests were statistically significant, then shortened and prolonged PRi groups were each compared to the normal PRi group. The level of statistical significance was set as *P* < 0.01 to account for multiple comparisons (normal versus prolonged PRi and normal versus shortened PRi).

Separate multivariable linear regression models were constructed to determine the associations with the PRi and LV measures and ejection fraction with the PRi fitted as a prolonged (>200 ms) or shortened (<120 ms) PRi compared to a normal PRi (120–200 ms). Linear regression models were then created with the dependent PRi examined as a continuous variable (per standard deviation unit or 25 ms). The Kolmogorov-Smirnov and the Shapiro-Wilk expanded tests were used to examine the assumption of a normal distribution for the LV measures. For each dependent variable, three regression models were examined. Model 1 adjusted for age, sex, race, height, and weight. Model 2 added site and heart rate to Model 1. Model 3 then added use of antihypertensive medications (ace inhibitor, angiotensin II antagonist, beta blocker, calcium channel blocker, and diuretics), systolic blood pressure, current smoking status, and presence of diabetes to Model 2. Potential covariates including total cholesterol and use of antiarrhythmic medications, digitalis preparations, cholesterol lowering mediations, or glucose lowering medications were not included in the final model because they were not associated with any change in the parameter estimates for PRi after adjustment for all variables in Model 3. The multivariate linear regression models were then repeated after excluding 967 participants using medications that may slow AV nodal conduction (beta-blockers, calcium channel blockers, digoxin, and/or antiarrhythmic medications).

To explore whether the associations between PRi and LV mass, LV mass index, LV dimensions, and ejection fraction were modified by race/ethnicity, interaction terms for race × PRi were fitted in the models with all covariates and with all participants. If the interaction term reached a statistical significance level of *P* < 0.1, then interaction terms for each nonwhite race × PRi were included in a model with all covariates. Race-specific associations for the association between the PRi and LV measures were obtained via linear combinations of the model's main PRi coefficient and the race × PRi interaction coefficient.

## 3. Results

Overall, mean age was 61.5 years (10.1) and 47.6% were male. Race/ethnicity was white in 39.1%, Chinese in 13.1%, African-American in 25.7%, and Hispanic in 22.2%. The PRi ranged from 88 to 308 ms with a median value of 162 ms and mean value of 165 ms (standard deviation 25). Of the 4962 subjects in the analysis cohort, 49 (1.0%) had a PRi < 120 ms, 4503 (90.7%) had a PRi from 120–200 ms, and 410 subjects (8.3%) had a prolonged PRi (>200 ms). Individuals with a PRi > 200 ms were older, taller, heavier, and more likely to be male (Table 1). Both heart rate and systolic blood pressure were significantly higher and QRS duration was significantly longer among participants with a PRi > 200 ms compared to those with a PRi 120–200 ms. Participants with a prolonged PRi were more likely to be using calcium channel blockers, beta-blockers, and diuretics compared to participants with a PRi 120–200 ms (Table 2).

Table 3 shows the mean values of LV measures and ejection fraction by presence of a PRi < 120 ms, 120–200 ms, or > 200 ms. Compared to participants with a PRi 120–200 ms, participants with a PRi > 200 ms had higher mean LV stroke volume indexed for body surface area (48.6 (10.4) versus 46.6 (8.8); *P* < 0.001), higher LV end-systolic (22.9 mL/m^2^ (9.4) versus 21.2 mL (7.9); *P* < 0.001), and higher LV end-diastolic volumes indexed for body surface area (71.6 mL/m^2^ (16.1) versus 67.8 mL (13.2); *P* < 0.001). A prolonged PRi was also associated with higher LV mass (163.3 g (46.3) versus 143.7 g (38.4); *P* < 0.001) and LV mass indexed for body surface area (83.9 g/m^2^ (19.5) versus 71.4 (15.9) g/m^2^) compared to a PRi 120–200 ms (Table 3). A shortened PRi was associated with significantly lower levels of LV stroke volume indexed for body surface area and LV end-diastolic volume indexed for body surface area compared to a PRi 120–200 ms (Table 3). No significant difference was noted in ejection fraction across the PRi groups.

**Figure 1:**
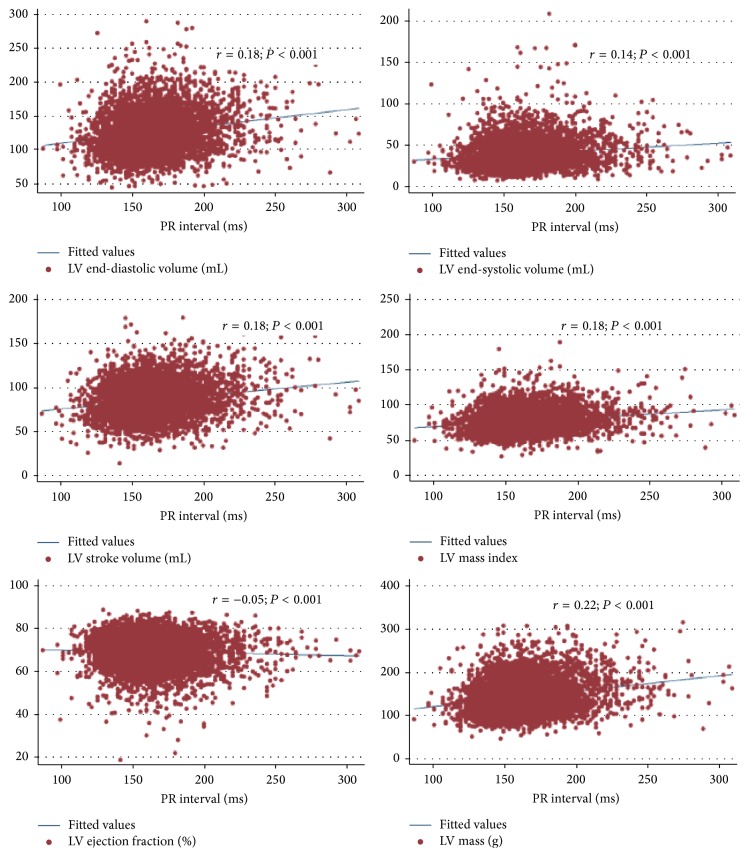
Scatterplots of PR interval by left ventricle dimensions, left ventricle mass index, and ejection fraction.

Figure 1 shows the scatterplots and Spearman's rank correlation coefficients for PRi and the LV measures and LV ejection fraction. The correlation between PRi and the LV measures ranged from as low as −0.05 (*P* < 0.001) for LV ejection fraction to as high as 0.22 (*P* < 0.001) for LV mass. In the regression analyses, presence of a PRi > 200 ms was associated with significantly higher LV stroke volume (4.07 mL; 95% CI 2.41, 5.72), higher LV mass (5.51 g 95% CI 2.89, 8.13) and LV mass index (2.56 g/m^2^; 95% CI 1.18, 3.94), and higher LV end-systolic (2.72 mL; 95% CI 1.25, 4.20) and end-diastolic volumes (6.80 mL; 95% CI 4.32, 9.28) compared to presence of a PRi 120–200 ms after adjustment for all covariates (Table 4). Presence of a PRi < 120 ms was associated with significantly lower LV stroke volume (−4.78 mL; 95% CI −9.17, −0.39) compared to a PRi 120–200 ms after adjustment for all covariates. However, compared to a PRi 120–200 ms, no significant association was noted between a PRi < 120 ms and LV mass, LV mass index, LV end-systolic or end-diastolic volume, or ejection fraction in any of the models (data not shown).

As a continuous variable, every standard deviation unit (25 ms) increment in PRi was associated with higher LV stroke volume (2.00 mL 95% CI 1.52, 2.48), higher LV mass (3.08 g; 95% CI 2.30, 3.86) and LV mass index (1.36 g/m^2^; 95% CI 0.96, 1.76), and higher LV end-systolic (1.31 mL; 95% CI 0.88, 1.73) and end-diastolic (3.31 mL 95% CI 2.58, 4.03) volumes after adjustment for all covariates. In the sensitivity analyses which excluded participants using medications that may slow AV nodal conduction, every incremental standard deviation unit increase in PRi remained associated with significantly higher LV stroke volume (1.19 mL; 95% CI 0.72, 1.67), LV mass (2.87 g; 95% CI 2.06, 3.68) and LV mass index (1.00 g/m^2^; 95% CI 0.56, 1.44), and higher LV end-systolic (1.19 mL; 95% CI 0.72, 1.67) and end-diastolic (2.87 mL; 95% CI 2.06, 3.67) volumes after adjustment for all covariates. No significant association was noted between the PRi and ejection fraction (−0.20; 95% CI −0.43, −0.03) after adjustment for all covariates in the sensitivity analyses.

The interaction term for race × PRi fitted in the model with all participants and adjusting for all covariates was not significant in models with LV stoke volume, LV end-systolic volume, or LV end-diastolic volume as the dependent variable. However, the interaction term for race × PRi did meet statistical significance in the model with LV mass and LV mass index as the dependent variable (*P* < 0.001). Among whites, every standard deviation unit increment in PRi was associated with a 1.33 g (95% CI 0.26, 2.41) higher LV mass after adjustment for all covariates. Compared to whites, the association between every standard deviation unit increment in PRi and LV mass was 2.29 g higher in African Americans (95% CI 1.06, 3.51) and 2.96 g higher in Hispanics (95% CI 1.33, 2.96). No significant difference in the association between PRi and LV mass was noted between Asians and whites after adjustment for all covariates (−0.05; 95% CI −0.33, 0.43). Similar results were noted for LV mass index (data not shown).

## 4. Discussion

This study demonstrates that a prolonged PRi is associated with significantly higher LV stroke volume and LV mass and LV mass index but not ejection fraction. This study also shows that that the associations between the PRi and LV mass and LV mass index differ by race/ethnicity with stronger associations noted among African American and Hispanic adults. In the CARE-HF trial [14], a prolonged PRi was one of three independent predictors of cardiovascular hospitalization and mortality in patients with severe heart failure. In the Health, Aging, and Body Composition study, a cohort of 2722 white and black adults with a mean age of 74 years at baseline, every 29 ms higher PRi was associated with a 13% increase in the 10 year risk of developing heart failure (95% CI 1.02, 1.25). A prolonged PRi was also associated with a heightened risk for the combined endpoint of heart failure or cardiovascular mortality (HR 1.61; 95% CI 1.02, 2.54) in the Heart and Soul Study, a cohort of adults with stable coronary artery disease [8]. It is possible that associations between a prolonged PRi and future risk of heart failure are mediated, at least in part, by a prolonged PRi reflecting higher LV mass index [7, 17, 18]. Although a few studies that examined associations between the PRi and cardiovascular outcomes adjusted for left ventricular hypertrophy, residual confounding may have existed due to lack of direct measures of LV mass [1, 10, 19, 20].

A prolonged PRi has also been linked with increased risk for atrial fibrillation [1, 21]. Using data from the Framingham Heart Study, Schnabel et al. found that the PRi adds discriminatory value to a 5-year risk prediction model for atrial fibrillation, which also included demographic data, systolic blood pressure, use of blood pressure lowering medications, and presence of heart failure [20]. Although this risk prediction model was validated in both whites and African Americans [19], other studies have not consistently demonstrated a significant association between the PRi and risk for atrial fibrillation [21]. Inconsistent associations have also been noted between PRi and mortality [6, 7, 22, 23]. These inconsistent associations have been attributed to differences in the level of contribution of *P* duration to the length of the PRi within and across populations [22].

While the MESA study was limited by lack of information on left atrial dimensions, higher LV mass could potentially link a prolonged PRi with increased future risk for atrial fibrillation [24–27]. The hypothesized mechanistic link between elevated LV mass and atrial fibrillation is supported by studies demonstrating strong associations between long standing hypertension and increased risk for atrial fibrillation [28–30]. In the MESA study, individuals with a PRi > 200 ms had higher systolic blood pressure and were more likely to be using antihypertensive medications compared to those with a PRI ≤ 200 ms. However, the role of elevated LV mass for risk of atrial fibrillation likely depends on its interaction with other factors such as ventricular wall stress, ischemia, scar tissue, and electrolyte abnormalities [24].

Our study noted that the association between PRi and LV mass index differs by race. Few studies have explored racial differences in the association between the PRi and cardiovascular risk factors and outcomes. The Atherosclerosis Risk in Communities (ARIC) study included 14, 433 adults (25% African American and 75% white) and in this cohort both obesity and hypertension, strong risk factors for both heart failure and atrial fibrillation, were associated with a prolonged PRi and associations were stronger among African Americans compared to whites [31]. In contrast, the Health, Aging, and Body Composition study did not find differences in risk of heart failure or atrial fibrillation between African American and white adults [7]. Shulman et al. examined the PRi among 50,870 adults followed for a mean of 3.7 years and 5,1999 developed atrial fibrillation. While atrial fibrillation risk by presence of a prolonged PRI was significantly higher among whites compared to Hispanics and African Americans, a significant increase in the risk for atrial fibrillation was noted at lower PRi levels for both Hispanic and African Americans as compared to whites. Thus, it is likely that the association between the PRi and atrial fibrillation, and perhaps other cardiovascular outcomes, differs by race [1].

The strengths of this study include the inclusion of adults from four different racial/ethnic groups and standardized measures of multiple measures of LV dimensions and ejection fraction by MRI. Because all MESA participants were free of clinical cardiovascular disease at baseline; the findings of this study may not be applicable to individuals with established clinical cardiovascular disease such as heart failure. Information on atrial dimensions and electrolyte abnormalities was not available. The associations between PRi and LV dimensions were not strong and could be due to residual confounding. The cross-sectional design of this study precludes determination of temporal associations.

In conclusion, the PRi is associated with measures of LV stroke volume and LV mass but not ejection fraction. The association between a prolonged PRi and cardiovascular outcomes including heart failure and atrial fibrillation noted in previous studies may be due in part to a prolonged PRi indicating higher LV mass.

## Figures and Tables

**Table 1:** Characteristics by presence of a prolonged PR interval (PRi).

Variable	PRi < 120 ms (*n* = 49)	PRi 120–200 ms (*n* = 4503)	PRi > 200 ms (*n* = 410)	*P* value
Age (years)	58.0 (0.1)	61.2 (10.0)	65.7 (10.1)^*∗*^	<0.001
Male (%)	28.6^*∗*^	46.4	62.7^+^	<0.001
Race/ethnicity				<0.001
White (%)	57.1^*∗*^	38.6	39.3	
Black (%)	14.3^*∗*^	24.9	36.0	
Hispanic (%)	16.3^*∗*^	23.1	14.3^+^	
Chinese (%)	12.2	13.4	10.4	
Waist circumference (cm)	92.7 (13.5)	96.3 (13.3)	99.3 (12.5)^+^	<0.001
Height (cm)	164.4 (8.1)	166.6 (9.9)	170.1 (9.8)	<0.001
Weight (kg)	70.3 (14.2)^*∗*^	76.6 (16.1)	82.5 (16.1)^+^	<0.001
Current smoker (%)	16.3	13.0	10.3	NS
Systolic blood pressure (mmHg)	122.6 (22.1)	125.1 (21.2)	130.0 (21.0)	<0.001
Diastolic blood pressure (mmHg)	72.0 (10.8)	71.8 (10.3)	72.8 (10.0)	0.2
Diabetes (%)	8.2	10.8	12.9	0.5
Heart rate (beats/minute)	65.2 (9.2)	63.1 (9.4)	59.9 (9.2)^+^	<0.001
QRS duration (ms)	93.3 (13.5)	92.9 (13.4)	97.7 (16.0)^+^	<0.001
PR interval (ms)	111.6 (6.6)	160.8 (17.7)	218.2 (19.7)^+^	<0.001

^+^ *P* < 0.001 compared to PRi interval 120–200 ms. ^*∗*^ *P* < 0.01 compared to PRi interval 120–200 ms.

**Table 2:** Baseline medication use by presence of a prolonged PR interval (PRi).

Medication type	PRi < 120 ms (*n* = 49)	PRi 120–200 ms (*n* = 4503)	PRi > 200 ms (*n* = 410)	Overall *P* value
ACE inhibitor (%)	6.1	11.6	16.8^*∗*^	0.003
Angiotensin 2 antagonist (%)	2.0	4.7	6.5	0.2
Beta-blocker (%)	4.1	8.5	15.1^+^	<0.001
Calcium channel blocker (%)	2.0	11.3	18.5^+^	<0.001
Antiarrhythmic medication (%)	0	0.04	1.7^*∗*^	0.001
Digitalis preparation (%)	0	0.3	1.0	0.07
Diuretic medication (%)	0^*∗*^	11.6	19.2^+^	<0.001
Cholesterol medication (%)	10.2	15.4	18.2	0.2
Any hypertension medication (%)	12.2^*∗*^	34.0	51.6^+^	<0.001

^+^ *P* < 0.001 compared to PRi 120–200 ms; ^*∗*^ *P* < 0.01 compared to PRi 120–200 ms.

**Table 3:** Left ventricle (LV) ejection fraction and measures by PR interval (PRi).

Variable	PRi < 120 ms (*n* = 49)	PRi 120–200 ms (*n* = 4552)	PRi > 200 ms (*n* = 410)	Overall *P* value
LV mass (g)	130.6 (38.5)	143.7 (38.4)	163.5 (46.3)^+^	<0.001
^†^LV mass index (g/m^2^)	73.5 (16.5)	71.4 (15.9)	83.9 (19.5)^+^	<0.001
^†^LV end-systolic volume (mL)	20.6 (10.4)	21.2 (7.9)	22.9 (9.4)^+^	<0.001
^†^LV end-diastolic volume (mL)	64.2 (15.2)^*∗*^	67.8 (13.2)	71.6 (16.1)^+^	<0.001
^†^LV stroke volume (mL)	43.6 (9.0)^*∗*^	46.6 (8.8)	48.6 (10.4)^+^	<0.001
LV ejection fraction (%)	68.6 (8.3)	69.1 (7.4)	68.5 (7.8)	0.3

Data shown as mean (standard deviation).

^+^ *P* < 0.001 compared to PRi 120–200 ms; ^*∗*^ *P* < 0.01 compared to PRi 120–200 ms.

^†^Indexed for body surface area [16].

**Table 4:** Multivariable adjusted differences in LV measures and ejection fraction by presence of a prolonged PR interval (>200 ms) versus PR interval 120–200 ms.

LV measures	Model 1 *β* (95% CI)	Model 2 *β* (95% CI)	Model 3 *β* (95% CI)
LV mass (g)	7.17 (4.41, 9.94)	6.13 (3.38, 8.89)	5.51 (2.89, 8.13)
^†^LV mass index (g/m^2^)	3.57 (2.11, 5.03)	2.91 (1.45, 4.36)	2.56 (1.18, 3.94)
LV end-systolic volume (mL)	2.55 (1.09, 4.01)	2.56 (1.09, 4.04)	2.72 (1.25, 4.20)
LV end-diastolic volume (mL)	8.21 (5.69, 10.73)	6.93 (4.46, 9.41)	6.80 (4.32, 9.28)
LV stroke volume (mL)	5.67 (3.94, 7.39)	4.37 (2.71, 6.03)	4.07 (2.41, 5.72)
LV ejection fraction (%)	0.14 (−0.57, 0.85)	−0.12 (−0.83, 0.59)	−0.26 (−0.97, 0.45)

Model 1 adjusted for age, sex, race, height, and weight. Model 2 adds heart rate and site to Model 1.  Model 3 adds systolic blood pressure, use of antihypertensive medications, current smoking status, and diabetes to Model 2.

^†^LV mass indexed for body surface area [16].
